# Wild bees occupy temporally stable pollen nutritional niches

**DOI:** 10.1007/s00442-026-05936-z

**Published:** 2026-07-23

**Authors:** Anthony D. Vaudo, Jillian A. Luthy, Eva Lin, Sonja K. Glasser, Anne S. Leonard

**Affiliations:** 1 Rocky Mountain Research Station, USDA Forest Service, Moscow, ID 83843 USA; 2 Department of Biology, University of Nevada Reno, Reno, NV 89557 USA; 3 Department of Biology, University of Massachusetts Amherst, Amherst, MA 01003 USA

**Keywords:** Bee nutritional ecology, Pollen nutrition, Plant-pollinator interactions, Foraging behavior, Floral rewards

## Abstract

As bees’ primary source of protein and lipids, pollen is a nutritional resource key to the persistence of wild populations. Because bee taxa may differ in their pollen macronutrient requirements, and pollen is a limited resource that varies chemically across plants, nutritional niches may shape bees’ ecological interactions. To explore whether members of bee communities occupy pollen nutritional niches, we analysed the protein and lipid content of pollen collected by abundant bee subgenera (*Nomadopsis*, *Melanosmia, Eumelissodes, Pyrobombus*) and observed their pollen-collecting visitation to plant species at a single Sierra Nevada meadow across three years. We compared pollen nutrition and host plant visitation among subgenera, and at the species level within *Pyrobombus.* Subgenera exhibited clear and temporally consistent separation of pollen load macronutrition each year, achieved by either host-plant fidelity or flexibility when foraging. *Pyrobombus* species shared a nutritional niche, potentially creating competition for pollen macronutrients among species relieved by their taxonomic flexibility. These results offer some of the first evidence that nutritional niches among wild bee taxa are annually stable and demonstrate how different taxa may forage to achieve nutritional consistency. Relevant to bee conservation, our findings emphasize that a nutritionally diverse array of plants may be required to support wild bee communities.

**Supplementary Information:**

The online version contains supplementary material available at 10.1007/s00442-026-05936-z.

## Introduction

The pollen that bees collect varies widely in its nutritional value across plant taxa (Roulston and Cane 2000; Ruedenauer et al. 2019; Vaudo et al. 2020, 2024a; Chau and Rehan 2024; Stephen et al. 2024). Within a co-flowering plant community, for example, pollens from different species present foraging bees with a nutritionally diverse array of options, as some species’ pollen is relatively richer in protein, and others offer richer sources of lipids (Vaudo et al. 2024a). Given that both these macronutrients can impact bee health and reproduction (reviewed in Vaudo et al. 2020), understanding whether and how pollen chemistry structures community interactions has emerged as a new question in pollination ecology, relevant to both uncovering the nutritional drivers of plant-pollinator interactions and informing their conservation (Vaudo et al. 2015; Filipiak 2018; Parreño et al. 2022).

In support of the idea that differences in bee nutritional needs can shape patterns of pollen host use, we recently found that across plant species from multiple sites in the Eastern Sierra/Great Basin (CA and NV USA), variation in pollen protein and lipid content predicted patterns of bee visitation: plants with nutritionally similar pollens (i.e., similar protein:lipid ratios) tended to have similar communities of bee visitors (Vaudo et al. 2024a). This work and that of others supports the “pollen nutritional niche” hypothesis (Parreño et al. 2022; Vaudo et al. 2024a; Bain et al. 2025) wherein the nutritional needs of a given bee taxon predict its use of a subset of available pollen host plants based on their nutritional value. When foraging, bee taxa could either specialize on hosts that provide pollen whose chemistry aligns with their nutritional needs, or forage more generally to combine nutritionally complementary pollens from different plants to achieve a balanced intake target (Machovsky-Capuska et al. 2016; Vaudo et al. 2024a). Via either mechanism, a basic prediction of the nutritional niche hypothesis is that bees should show consistency across both space (e.g. when foraging from different plant assemblages) and time (e.g. from year to year) in the macronutrient content of the pollen they collect. Alternatively, if bees routinely reshuffle the macronutrient profile of the pollen they collect, this would suggest either that the choice of focal nutrients bears reconsideration, or perhaps more broadly that nutritional niches are not a durable concept for understanding whether nutrition organizes community interactions between bees and plants across space and time.

*Manipulative* research on focal species suggests that bee nutritional targets may differ between species (e.g., Barraud et al. 2022). But, the basic question of whether wild populations of bees consistently differ in their collection of pollen macronutrients is surprisingly open because most studies either indirectly estimate bees’ collected pollen nutrition from the plant species they visit (Vaudo et al. 2024a; Bain et al. 2025) or quantify the nutritional value of bee-collected pollen within a single sampling period/season. At any one location from year to year, we might expect foraging bees to cope with several nutritional perturbations. Most obviously, changes to the plant community (gains, losses, phenological shifts) affect what pollen is available for bees to collect. More subtly, resources may be available but altered, as environmental variables such as temperature and water availability can alter the protein and lipid content of pollen (Russo et al. 2019; Descamps et al. 2021a, b, c; Vaudo et al. 2024b). Even if the identity, quantity, and quality of resources are constant, consumers’ needs could vary based on the stage in their life cycle or biotic/abiotic stressors (Dolezal and Toth 2018; Hendriksma et al. 2019; Kraus et al. 2019; Vanderplanck et al. 2019; Carnell et al. 2020; Parreño et al. 2022; Crone and Grozinger 2021). Therefore, evidence that bees show consistency in pollen macronutrient collection over years would support the idea that nutritional niches are strong drivers of foraging patterns, despite these potential sources of variation.

Assessing the temporal stability of nutritional niches might also shed light on our understanding of interactions among members of bee communities. Namely, several lines of evidence suggest that bees within a community do compete for pollen (Brosi and Briggs 2013; Cane and Tepedino 2017; Wignall et al. 2020; Page and Williams 2023), although these dynamics are rarely considered from a nutritionally explicit perspective (but see Page et al. 2024). If competition for nutritionally similar pollen resources occurs, we might find several patterns in a stable community without recent disturbance. First, it is possible that competition might preclude bees with similar nutritional niches from co-occurring. Alternatively, we might find evidence that competition has resulted in bees with similar nutritional needs partitioning foraging effort across taxonomically distinct but nutritionally similar host plants (Behmer and Joern 2008; Machovsky-Capuska et al. 2016; Johnson and Bronstein 2019; Pasquali et al. 2025). Finally, if competition is ongoing, we could find the opposite pattern: bees with similar nutritional needs might visit similar suites of host plant species. Describing which, if any, of these patterns wild bee populations consistently demonstrate can help set the stage for further manipulative investigations of whether and how members of bee communities compete for shared pollen nutrients.

To explore the existence of pollen nutritional niches among bee taxa, we chemically analyzed the pollen loads collected by wild bees, and recorded their foraging patterns in a single Sierra Nevada Mountain meadow across three years. The site’s high floral abundance but low species diversity, combined with a short growing season, offered an ideal context in which to study how focal abundant bee taxa organized their use of available pollen resources. Specifically, we compared patterns of floral visitation and pollen load macronutrient content, to ask (Q1) whether abundant bee taxa differ in patterns of floral visitation that give rise to annual differences in pollen load nutrition; (Q2) whether bee taxa with relatively similar pollen load nutrition achieved this by visiting taxonomically similar vs. different plant assemblages, and (Q3) how stable these nutritional and foraging patterns were across three summers.

## Methods

### Sample collection

**Fig. 1:**
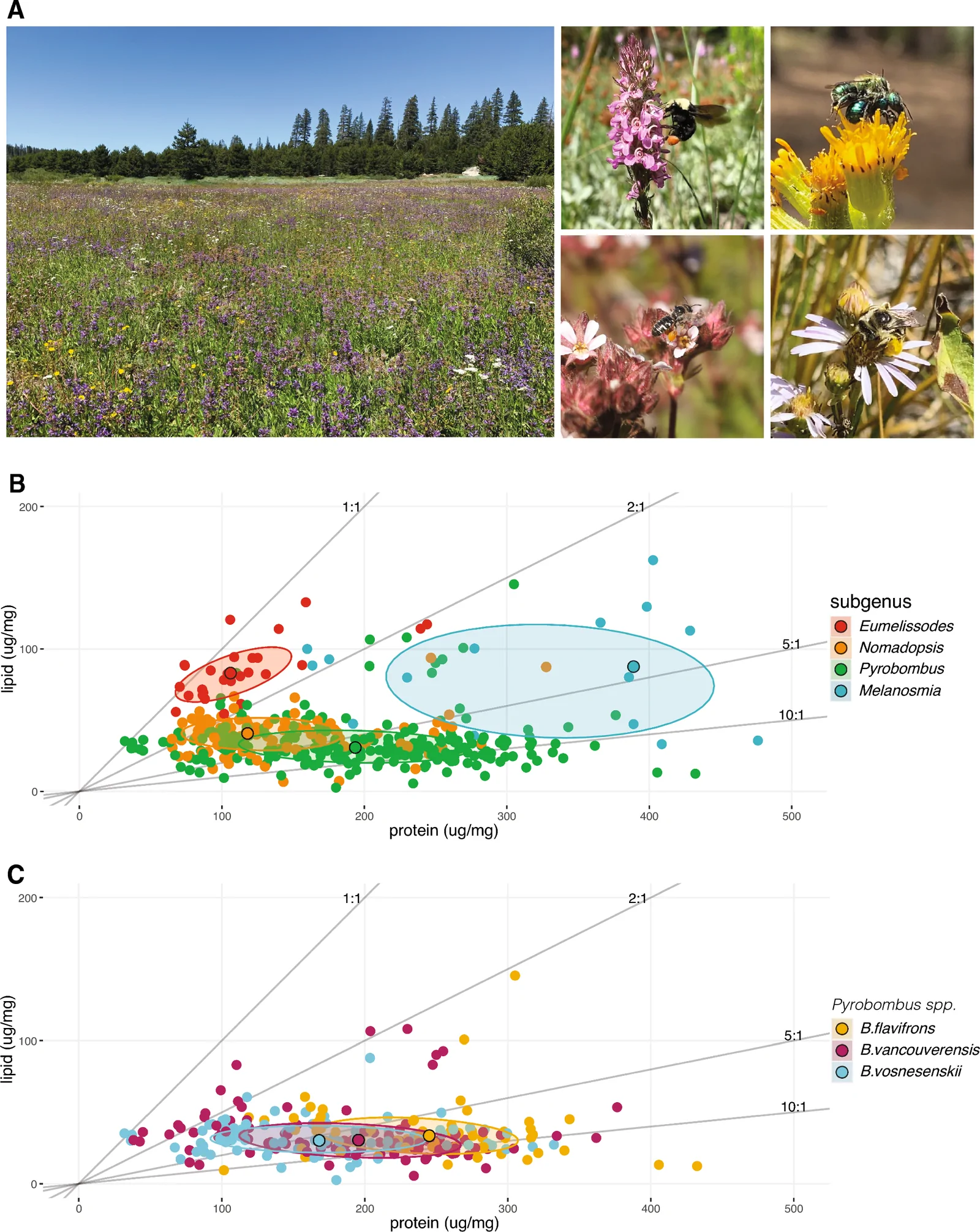
**A**) Study site and bees represented in this study. Left: The meadow at Lake Van Norden, CA. Middle top: *Bombus (Pyrobombus) vosnesenskii*; top right: *Osmia (Melanosmia)* sp.; bottom right: *Melissodes (Eumelissodes)* sp.; middle bottom: *Calliopsis (Nomadopsis) edwardsii*. **B**) Bee subgenera and **C**) *Pyrobombus* spp. pollen loads in nutritional space: Markers = individual bee pollen load (color = subgenus or *Pyrobombus* spp.); black outlined markers represent subgenus or species median values surrounded by 50% CI ellipses. Labeled diagonals = P:L ratios

We collected female bees foraging in a meadow at Lake Van Norden in the Sierra Nevada Mountain range (39.31355, -120.36081, ~ 2,050 m elevation, ~ 356 hectares/880 acres; Fig. 1A) during peak blooming times in mid-late season (seven days in between 8-Aug-2019 – 20-Aug-2019, seven days between 7-Jul-2020 – 20-Aug-2020, and five days between 21-Jun-2021 – 20-Jul-2021). Flowering phenology varied across years, perhaps in line with environmental variables (e.g., mean daily temperature of sampling dates was lowest in 2019, and highest in 2021, Table S1), yet the plant community exhibited little turnover in species composition across years or throughout the short growing season. We used an initial visual survey to focus our observations on the most abundant flowering plant taxa in the meadow, including the families Asteraceae, Fabaceae, Malvaceae, Montiaceae, Onagraceae, Orobanchaceae, Plantaginaceae, and Rosaceae (Table 1). Plants were identified to species by Herbarium Curator Arnold Tiehm of the University of Nevada Reno Museum of Natural History. We determined the pollen protein concentrations, lipid concentrations, and protein:lipid (P:L) values of each species in 2021 (Vaudo et al. 2024a) (Table 1). Within the meadow and along forest edges, we identified focal patches of each plant species that had at least ten individual plants in bloom (most had > 50) and recorded GPS waypoints for revisiting. Each day we moved among patches, netting all bees we observed actively collecting pollen or contacting flower anthers for 10 min at each plant species. Bees were immediately placed in individual centrifuge tubes, housed within an ice-filled cooler, then stored at -20 ℃ in the lab until further processing. In the lab, we further confirmed our samples represented only actively pollen-collecting bees by excluding males as well as females visually estimated to have scopa < 10% filled with pollen. We then used stainless steel insect pins to transfer pollen from individual bees’ scopae or corbiculae (hereafter referred to as “pollen loads”) into individual 2-mL centrifuge tubes, stored at -20 ℃.

**Table 1:** Focal plant species and pollen nutritional values (Vaudo et al. 2024a)

Order	Family	Species	protein (μg/mg)	lipid (μg/mg)	P:L
Asterales	Asteraceae	*Achillea millefolium*	91.0	101.6	0.9
Asterales	Asteraceae	*Arnica chamissonis*	116.2	102.3	1.1
Asterales	Asteraceae	*Oreostemma alpigenum*	78.6	65.6	1.2
Asterales	Asteraceae	*Solidago lepida*	98.0	85.6	1.1
Asterales	Asteraceae	*Symphyotrichum spathulatum*	90.7	70.7	1.3
Caryophyllales	Montiaceae	*Calyptridium umbellatum*	203.9	23.0	8.9
Fabales	Fabaceae	*Lupinus lepidus*	338.8	18.9	17.9
Lamiales	Orobanchaceae	*Pedicularis attollens*	398.0	13.8	28.9
Lamiales	Plantaginaceae	*Penstemon heterodoxus*	402.4	56.5	7.1
Malvales	Malvaceae	*Sidalcea glaucescens*	46.3	53.6	0.9
Malvales	Malvaceae	*Sidalcea oregana*	49.8	53.5	0.9
Myrtales	Onagraceae	*Chamerion angustifolium*	150.9	52.2	2.9
Rosales	Rosaceae	*Horkelia fusca*	160.6	45.5	3.5
Rosales	Rosaceae	*Potentilla gracilis*	168.1	64.7	2.6

After this, we pinned all bees and identified them to the lowest taxonomic level possible targeting the subgenus (or species level for *Bombus*) using the keys and nomenclature in (LaBerge 1956; Rozen 1958; Michener et al. 1994; Michener 2007; Koch et al. 2013; Ascher and Pickering 2020); and with input from Terry Griswold and Harold Ikerd (USDA-ARS) and James Strange (Ohio State University). Bee specimens were added to the collections of the University of Nevada, Reno Museum of Natural History or the Rocky Mountain Research Station’s Moscow Forestry Sciences Laboratory (specimen collection numbers provided in Table S1). We did not collect *Bombus* gynes, which were rarely observed in the meadow, and therefore only workers are included in the analysis.

### Analysis and sample sizes

We compared pollen load nutrition and pollen host plant visitation within and between years at two levels of taxonomic analysis: (Comparison 1) Among the four most frequently observed subgenera that had at least four pollen loads per year to analyze. These were *Calliopsis (Nomadopsis)* spp. (Hymenoptera: Andrenidae) (N = 107), *Osmia (Melanosmia)* spp. (Megachilidae) (N = 30), *Melissodes (Eumelissodes)* spp. (Apidae) (N = 28), and *Bombus (Pyrobombus)* spp. (Apidae) (N = 275; Fig. 1A; for sample sizes by year see Table S2). (Comparison 2) Within the *Pyrobombus* (bumble bee) subgenus for which we were able to collect the most observations and pollen loads. These were *Bombus flavifrons* (*B. flavifrons dimidiatus* color morph, N = 63)*, B. vancouverensis* (formerly *B. bifarius subsp. nearcticus*, N = 107), and *B. vosnesenskii* (N = 96; for sample sizes by year, see Table S3).

### Pollen load nutritional analysis

To minimize sampling bias, we centrifuged an individual pollen load to the bottom of its respective tube, mixed it with a small spatula, then divided it evenly into two separate 2 mL tubes, with up to 2 mg of pollen in each. We used the sample in one tube to analyze protein concentrations (via the Bradford assay) and the sample in the other tube to analyze lipid concentrations (via the sulfo-phospho-vanillin assay; Table S1). Methods for these assays are detailed in (Vaudo et al. 2024a); in a slight modification, we analyzed each pollen load sample once (rather than in triplicate) for protein and lipid content due to the small amount of pollen available. We then used this information to calculate individual P:L ratios for each pollen load (Table S1; Vaudo et al. 2018).

For both comparisons (1) and (2), we used a Bayesian modeling approach to compare the nutritional value of pollen loads collected across bee taxa using the R (CRAN) package *rethinking* (McElreath 2020). We standardized pollen nutrition (protein µg/mg, lipid µg/mg, or P:L ratios) using z-scores. For overall differences between taxa across all years, we used quadratic approximation with the function *quap* (SI Model 1). For between-year comparisons, we used adaptive priors for the distribution of pollen nutrients in each year using the function *ulam* which incorporates Hamilton chain Monte Carlo through Stan (Stan Development Team 2025) (SI Model 2). After obtaining posterior distributions, we used the function *link* to render posterior predicted means and 90% credible intervals (CI) to obtain predicted values of pollen nutrition for each bee taxon.

### Host plant foraging analysis

For both comparisons (1) and (2), we created visitation matrices for both overall (metanetwork) and within-year visitation records. Matrices used bee taxa or bee taxa-by-year as sites (rows) and plant family as species (columns) (Table S4). We opted to run our analyses at the plant family level because 1) there was an unequal number of plant species within each family that could skew interpretation of plant visitation and 2) plant species within each family offered pollen of similar nutritional value (Table 1) and are visited by taxonomically similar communities of bees (Vaudo et al. 2024a); however, running analyses at the species level produced qualitatively similar results (see Supplemental Figs S2–4, S6–8 and Tables S7–8). We used Principal Components Analysis (PCA) on the bee-year by plant family matrices to evaluate visitation differences in the package *vegan* (Oksanen et al. 2022). Because of unequal sampling time between years, and thus observed bee-plant interactions, we used the function *decostand* and *method* = *“normalize”* to normalize the data between rows. We used the function *rda* on the bee subgenus or *Bombus* spp. normalized matrices. To visualize clustering indicative of host plant associations, we plotted bee and plant taxa PC1 and PC2 scores. We then used the function *envfit* (where average bee collected pollen nutrition or bee taxa centroids are regressed against ordination components; SI Model 3) and *adonis2* (permutational multivariate analysis of variance of average bee collected pollen nutrition or bee taxa against distance matrices of visitation data; SI Model 4) to determine if a) bee taxa differed in host plant use, and b) if the average protein, lipid, or P:L ratios of bee pollen loads were associated with differences in bee visitation to plant-taxa. Significant values at *P* < 0.05 would indicate a) that bee taxa differ in their primary (pollen) host-plant use, and b) that pollen load nutrition is associated with different (pollen) host-plant use patterns.

To further examine between-taxa foraging strategies, we obtained the network indices *effective partners* and *d’* for comparing bee taxa levels of specialization to plant taxa both across and within years using the function *specieslevel* in the package *bipartite* (Dormann 2011). Low *effective partners* (diversity of interactions) and high *d'* (specialization of a species based on selectivity compared to partner availability) values indicate more specialized (less generalized) patterns of floral visitation.

## Results

### Among bee subgenera, pollen nutrition was relatively distinct, and these differences were consistent across years

**Fig. 2:**
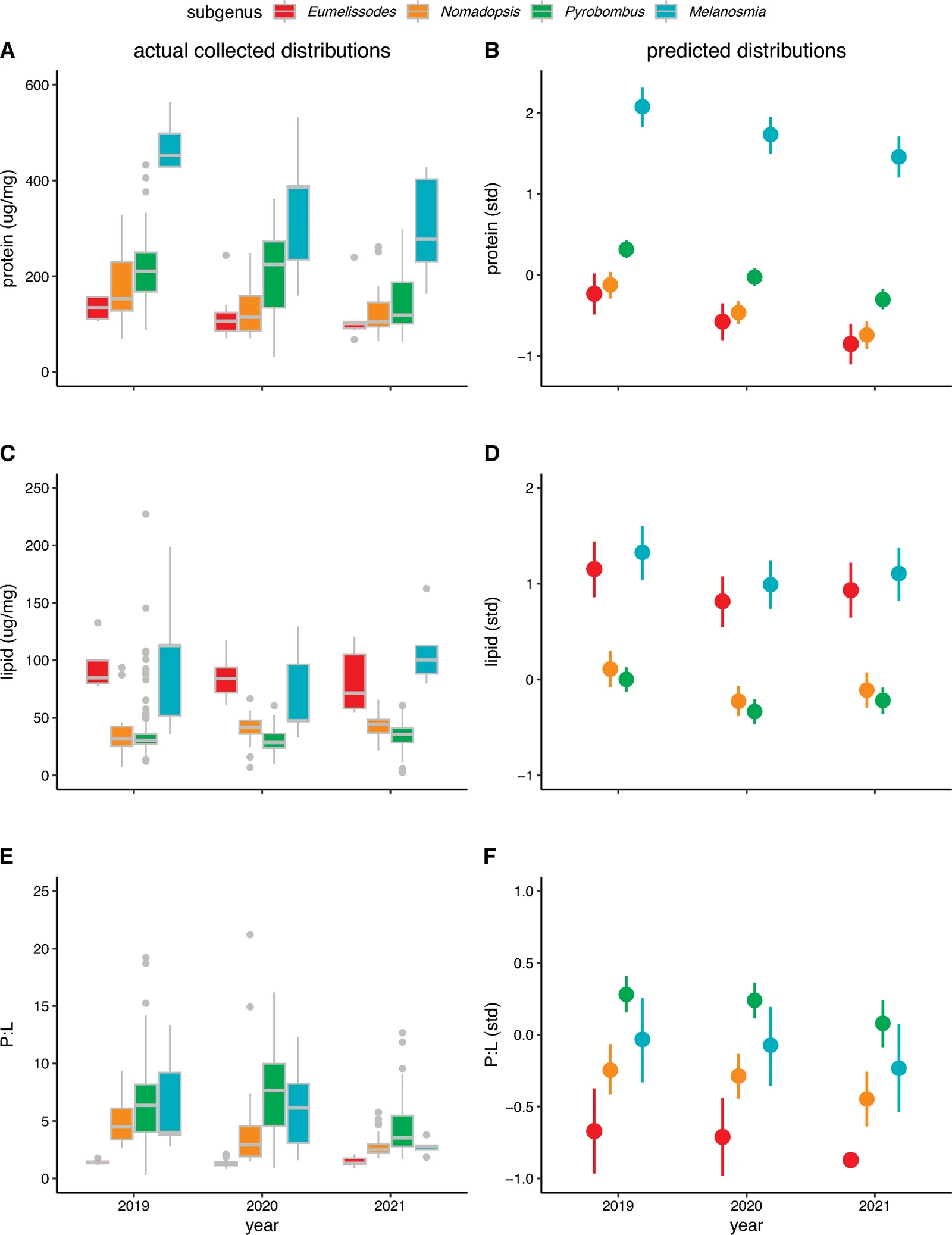
Bee subgenus pollen nutrition distributions in each year. Boxplots (**A, C, and E**) are actual distributions with median, 1st and 3rd quartiles and whiskers 1.5*IQR. Panels **B, D, and F** are Bayesian posterior predicted mean and 90% credible intervals, indicating predicted values of nutritional composition of each bee taxa in each year

Overall, the focal bee subgenera occupied different regions of nutritional space (Fig. 1B), although there was individual-level variation leading to some overlap in the nutrient concentration of pollen loads. Nutritional patterns among subgenera remained consistent for all three sampling years, especially when evaluating posterior predictions from the Bayesian analyses (Fig. 2, Table S5C–D). Protein concentrations generally decreased from 2019 to 2021, while lipid content was comparatively stable. Thus, P:L values of *Nomadopsis*, *Pyrobombus,* and *Melanosmia* tended to decrease from 2019 to 2021. Yet, subgenera maintained their relative positions in nutritional space: 1) *Melanosmia* collected the highest protein, followed by *Pyrobombus*, while *Nomadopsis* and *Eumelissodes* shared the lowest protein values (Fig. 2A–B). 2) *Melanosmia* and *Eumelissodes* collected the highest lipid values, while *Nomadopsis* and *Pyrobombus* collected the lowest (Fig. 2C–D). 3) *Pyrobombus* collected the highest P:L pollen, followed by *Melanosmia*, then *Nomadopsis*, and finally *Eumelissodes* (Fig. 2E–F, Table S5C–D). These trends among bee subgenera remained consistent when pooling pollen loads from all years (Fig S1, Table S5A–B).

### Dominant bee subgenera differed in host plant use within and across years

**Fig. 3:**
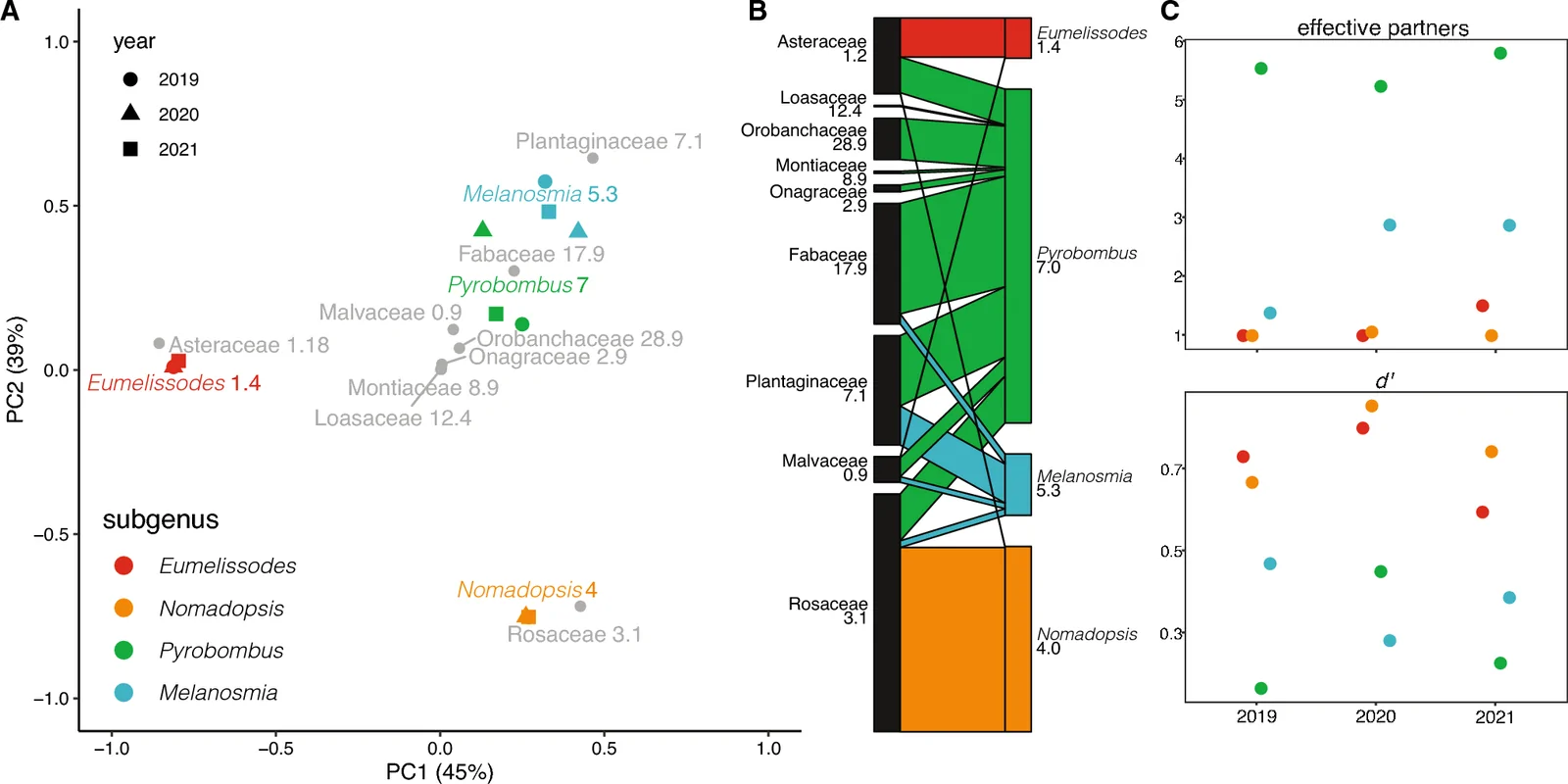
Associations between host plant visitation and pollen load nutrition across bee subgenera. **A**) Principal components (PCA) plots of bee visitation to plant families (color = bee subgenus; shape = year). Labels indicate average P:L values of pollen loads measured in this study, or floral pollen of plant taxa present at site (from Vaudo et al. 2024a). **B**) Interaction metanetwork across all three years. Width of bee taxa = relative numbers observed; width of plant taxa = relative numbers of visits observed; width of connectors = relative visitation frequency. Labels indicate average P:L values of pollen loads measured in this study, or floral pollen of plant taxa present at site. **C**) Indices of foraging specialization to plant families across years. Lower *effective partners* and higher *d’* values indicate higher levels of specialization

PCA plots of interactions between bee subgenera and pollen host plants showed yearly clustering and clear separation between bee subgenera due to consistent foraging patterns (Fig. 3A, S2). These analyses revealed close associations between *Eumelissodes* and Asteraceae plants, and *Nomadopsis* with Rosaceae plants, which were both separated from *Melanosmia* and *Pyrobombus. Pyrobombus* was more centered in the PCA plot because of its association with a variety of plant taxa, while *Melanosmia* was placed closely to its consistent associations with Plantaginaceae (*Penstemon*) (Fig. 3A–B, S2,3).

Thus, bee subgenera were significantly correlated to PC1 and PC2 of interaction matrices (*envfit*: *R*^*2*^ = 0.99, *P* = 0.001). Bee subgenus was also a significant predictor in the analysis of variance of distance matrices (*adonis*: *R*^*2*^ = 0.97, *P* = 0.003; Table S7A). The protein, lipid, and P:L content of pollen loads of bee subgenera were significantly correlated to variation in ordination space (*envfit*: protein: *R*^*2*^ = 0.64, *P* = 0.003, lipid: *R*^*2*^ = 0.71, *P* = 0.004, P:L: *R*^*2*^ = 0.55, *P* = 0.007) and were a significant predictor in analysis of variance of distance matrices (*adonis*: protein: *R*^*2*^ = 0.30, *P* = 0.001, lipid: *R*^*2*^ = 0.14, *P* = 0.018, P:L: *R*^*2*^ = 0.35, *P* = 0.001; Table S7A). Bee subgenera therefore differed in their pollen host plant use, and these differences reflected variation in the nutritional value of their pollen loads (Fig. 3A–B; see Discussion).

Beyond consistent differences in the taxonomic patterns of host use, bee subgenera also consistently differed in their degree of floral specialization. *Eumelissodes* and *Nomadopsis* were specialized to Asteraceae and Rosaceae respectively (Fig. 3C, S4, Table S8A–B). In contrast, *Melanosmia* and *Pyrobombus* were more generalized (visiting multiple plant families), yet maintained close associations to plants with higher P:L values (Fig. 3, S2,3, Table S8A–B; see Discussion). *Melanosmia* was moderate in these values, while *Pyrobombus* was the least specialized (Fig. 3C, S4, Table S8A–B).

### Bumble bee species exhibited slight differences and annually consistent trends in pollen nutrition

**Fig. 4:**
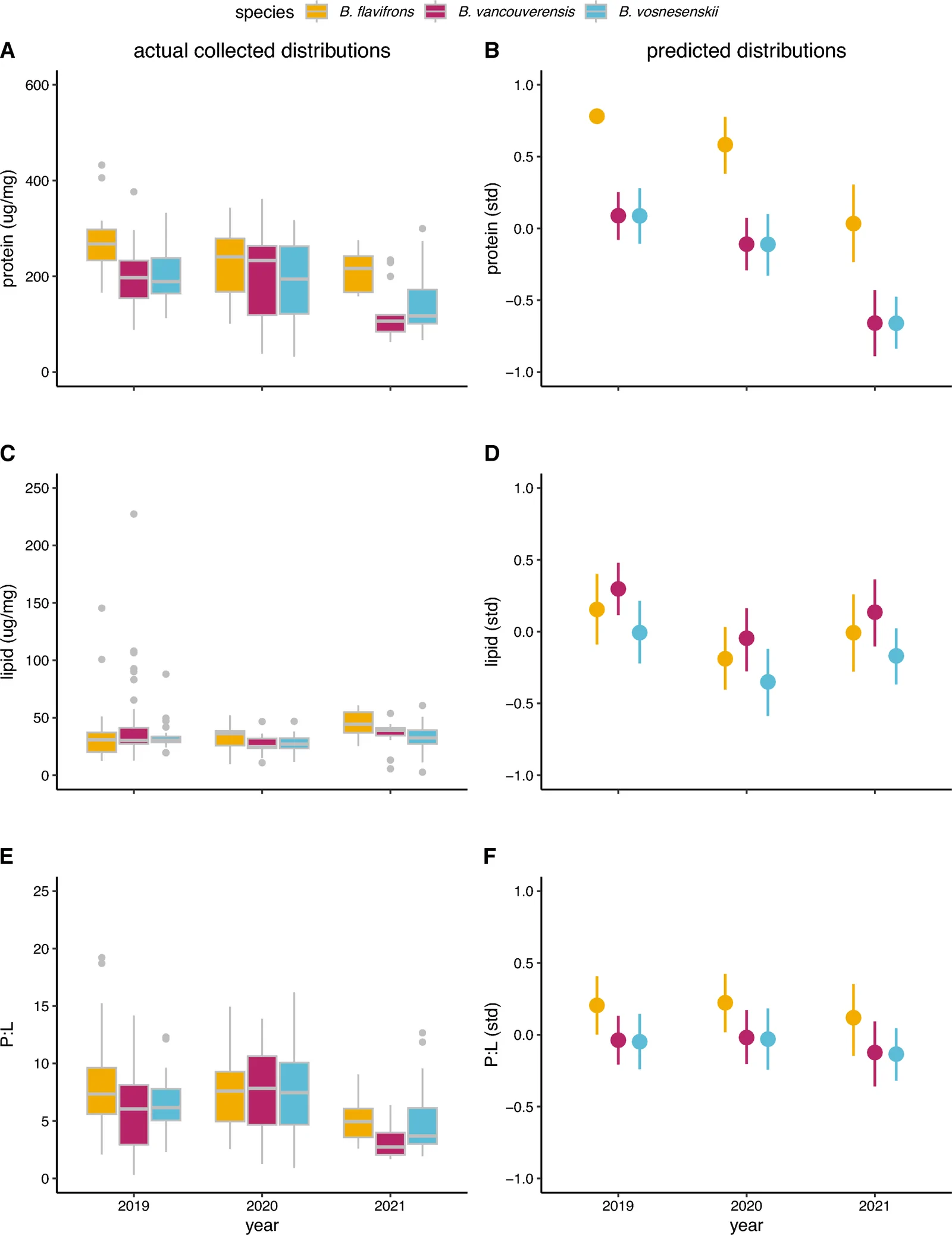
*Pyrobombus* species pollen nutrition distributions in each year. Boxplots (**A, C, and E**) are actual distributions with median, 1st and 3rd quartiles and whiskers 1.5*IQR. Panels **B, D, and F** are Bayesian posterior predicted mean and 90% credible intervals, indicating predicted values of nutritional composition of each bee taxa in each year

As generalist foragers occupying the highest P:L niche among focal subgenera, *Pyrobombus* pollen loads’ nutritional values were variable and overlapped substantially in nutritional space (Fig. 1C). Nonetheless, we discovered slight species-level differences. For example, the average protein content of pollen loads tended to decrease between 2019–2021 (Fig. 4A–B). Despite this, *B. flavifrons’* average pollen loads maintained their relatively highest protein content in comparison to *B. vancouverensis* and *B. vosnesenskii* (Fig. 4A–B, S5A–B, Table S6)*.* In contrast, pollen load lipid concentrations were relatively similar each year (Fig. 4C–D, S5C–D, Table S6). Because of the overall higher protein content collected by *B. flavifrons*, this species’ average P:L content was predicted to be slightly higher than *B. vancouverensis* and *B. vosnesenskii* which shared similar predicted pollen P:L (Fig. 4E–F, S5E–F Table S6).

### Bumble bee species shared host plants, but some species differed in primary hosts each year

**Fig. 5:**
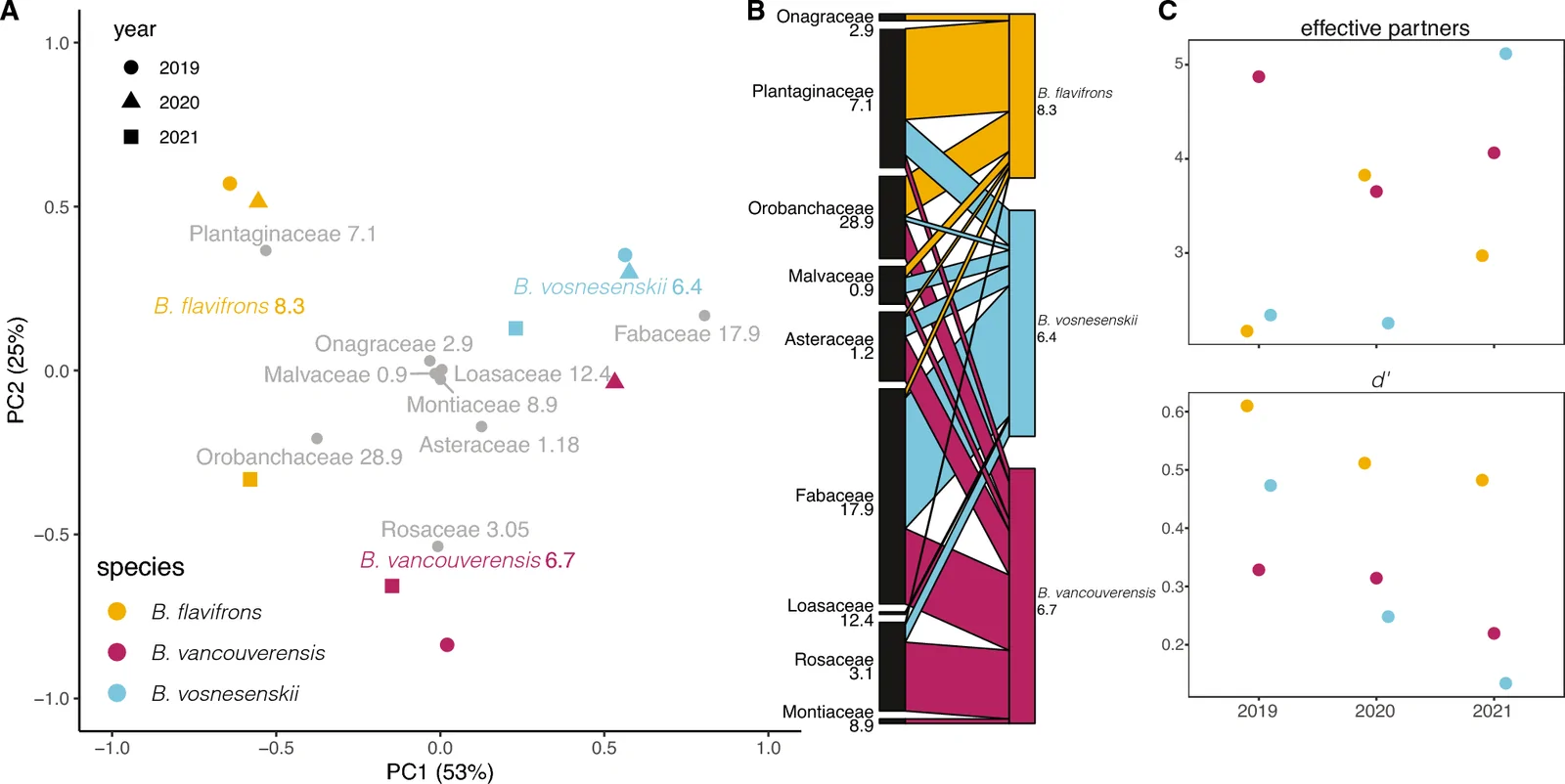
Associations between host plant visitation and pollen load nutrition among *Pyrobombus* species. **A**) Principal components (PCA) plots of bee visitation to plant families (color = species; shape = year). Labels indicate average P:L values of pollen loads measured in this study, or floral pollen of plant taxa present at site (from Vaudo et al. 2024a). **B**) Interaction metanetwork across all three years. Width of bee taxa = relative numbers observed; width of plant taxa = relative numbers of visits observed; width of connectors = relative visitation frequency. Labels indicate average P: L values of pollen loads measured in this study, or floral pollen of plant taxa present at site. **C**) Indices of foraging specialization to plant families across years. Lower *effective partners* and higher *d’* values indicate higher levels of specialization by each species

Although bumble bee species visited similar assemblages of pollen host plant species, they differed in their distribution of visits to these shared hosts. PCA plots show that *B. vosnesenskii* was tightly associated with Fabaceae (*Lupinus*) each year, whereas *B. flavifrons* and *B. vancouverensis* were more variable across years in host plant use (Fig. 5A,B, S6,7). *Bombus flavifrons* was frequently associated with Plantaginaceae (*Penstemon*) and Orobanchaceae (*Pedicularis*; Fig. 5A,B, S6,7). *Bombus vancouverensis* was more spread across the PCA plot with different major plant associations between years to *Lupinus, Penstemon, and Pedicularis*, and more associated with *Horkelia* and *Potentilla* (Rosaceae) than the other two species (Fig. 5A,B, S6,7).

Host-plant use among *Bombus* species resulted in separation along PC1 and PC2 (*envfit*; *R*^*2*^ = 0.70, *P* = 0.014, Table S7B). There was also moderate evidence that species was a significant predictor of variation in distance matrices (*adonis*; *R*^*2*^ = 0.63, *P* = 0.028; Table S7B). However, there was little to no evidence to suggest that differences in average nutritional values between bumble bee species’ pollen loads explained this variation in foraging strategies among plant families (*envfit*: protein: *R*^*2*^ = 0.47, *P* = 0.141, lipid: *R*^*2*^ = 0.57, *P* = 0.061, P:L: *R*^*2*^ = 0.41, *P* = 0.202; *adonis*: protein: *R*^*2*^ = 0.19, *P* = 0.065, lipid: *R*^*2*^ = 0.15, *P* = 0.204, P:L: *R*^*2*^ = 0.15, *P* = 0.389; Table S7B). These results indicate that although bumble bees occupy a high P:L niche, and *B. flavifrons* occupies the highest pollen protein niche, bumble bees were consistently partitioned by their different foraging frequencies to host plant species across years.

Within and across all years, *B. flavifrons* exhibited higher foraging specialization (*d'*) than *B. vancouverensis* and *B. vosnesenskii* (Fig. 5C, S8, Table S8C,D). However, the *Pyrobombus* species exhibited variable levels of *effective partners* across years (Fig. 5C, S8, Table S8C–D) indicating somewhat variable foraging among shared host plants between years.

## Discussion

Pollen functions as bees’ primary source of protein and lipids, and its acquisition is directly linked to reproductive performance and larval development (reviewed in Vaudo et al. 2015, 2020; Parreño et al. 2022). Growing recognition that different wild bee taxa have different nutritional requirements (e.g., P:L ratios) (Leonhardt and Blüthgen 2012; Vanderplanck et al. 2020; Barraud et al. 2022; Vaudo et al. 2024a) raises the obvious question of how these needs shape interactions among bees and their pollen host plants in ecological communities. If it is accurate and useful to describe bees as inhabiting pollen nutritional niches, we would expect to find evidence that within a community, 1) bee taxa collect nutritionally distinct resources at a given point in time, and 2) these differences in nutritional content persist across years. To our knowledge, the present study is one of the only to repeatedly characterize the pollen P:L ratios collected by wild bees from multiple lineages across multiple years.

In doing so, we found solid evidence of temporal (i.e., annual) consistency: the pollen loads analyzed from bee subgenera not only represented four different pollen P:L niches, but they also maintained their relative position in nutritional space across years. While manipulative work (i.e., restricting bees to different predetermined diets or host plants Tasei and Aupinel 2008; Vaudo et al. 2016a; Kraus et al. 2019; Treanore et al. 2019; Lawson et al. 2021; Barraud et al. 2022) would be required to demonstrate that these consistent P:L ratios we discovered in bees’ collected pollens maximize performance, our finding is an important piece of the puzzle because it shows that in real-world settings, bees (broadly) act as if they organize pollen foraging to achieve a consistent macronutrient target at the population level. Importantly, that target is not the same across bee subgenera, suggesting that the relative quality of pollen protein or lipid content is to a certain extent, taxon-dependent. Several other lines of evidence support this general idea—for example, specialist bee species do not develop well on non-host pollen (Praz et al. 2008); and, different bee genera have varying developmental and health outcomes when fed the same controlled diets differing in nutritional quality (Barraud et al. 2022). Recently, Bain et al. (2025) took a complementary approach, characterizing floral pollens and combining this information with visitation data to show that *Bombus* species are nutritionally consistent. Our data complement and extend this finding, showing that nutritional consistency is apparent in collected pollen loads across a taxonomically diverse range of bees.

For any non-monolectic taxa to achieve nutritional consistency across years may be challenging when abiotic factors, resource availability, and community interactions are likely to fluctuate; yet, our study suggests that bees do accomplish this, via multiple foraging strategies (Machovsky-Capuska et al. 2016). At the subgenus level, our two most specialized bee taxa (based on *d’* and *effective partners* network indices), *Melissodes (Eumelissodes)* and *Calliopsis (Nomadopsis)* showed consistency in the taxonomic identity of plant families they visited (suggesting oligolectic foraging strategies): year after year, they collected pollen almost exclusively from plant species within *Rosaceae* or *Asteraceae* (respectively), which were less frequently visited by the other common focal bee subgenera. Accordingly, the nutritional profile of their pollen loads closely matched that of floral pollen from their observed hosts, indicating that for these taxa, they maintained nutritional consistency via *taxonomic fidelity*. For example, *Nomadopsis* pollen loads’ P:L averaged 4, and its most frequently visited host *Horkelia* pollen’s P:L was 3.5; similarly, *Eumelissodes* pollen load P:L averaged 1.4, and its most frequently visited host *Symphyotricum* has a pollen P:L of 1.3 (Fig. 3, S2,3). Other bee subgenera (*Melanosmia, Pyrobombus*) maintained nutritional consistency via *taxonomic flexibility*, each foraging among combinations of plants that resulted in each respective subgenus collecting pollen loads with roughly the same protein and lipid content each year.

At sites such as ours with a relatively similar composition of flowering plants across years, the nutritional consistency of collected pollen that we observed might be a reasonable null expectation. However, it is not a foregone conclusion, as even in the face of a consistent host plant assemblage, shifts in floral chemistry and consumer need might be expected to generate some degree of variation in collected nutrition. Environmental variables that affect both bee and plant physiology may result in variation in nutritional chemistry among plants or floral choice among bees. For example, Descamps et al. (2021a,b) found that increased temperature altered floral pollen protein content (see also Vaudo et al. 2024b), and our prior findings indicated that bumble bees may increase protein collection on warmer days (Vaudo et al. 2018). We note that the protein content of bee-collected pollen possibly tracked local temperature, but a longer-term study would be needed to assess this relationship (see Table S1 for associated temperature data). Could bees at our site have been so constrained by the limited nutritional diversity of floral options that they were predestined to collect nutritionally similar resources year after year? We do not think so for three reasons: it is worth noting 1) that the pollens of multiple plant species (both within and across families) within the meadow shared similar nutritional qualities (Table 1), 2) the overall range of floral P:L values mirrors the nutritional diversity described in broader regional surveys (Vaudo et al. 2024a), and 3) the bees’ occupied nutritional niches align with those predicted across sites with significant floral species turnover (Vaudo et al. 2024a). Instead, our results suggests that at least some bees can and in fact do vary in their choice of host plant, foraging flexibly across years and combining visits to multiple resources to reach their macronutrient targets.

At any one time point, there are different ways that nutritional differentiation among bee taxa could arise. Most obviously, bee taxa could simply visit different host plants within the co-flowering community that vary in pollen macronutrient content—here again we note *Melissodes (Eumelissodes)* and *Calliopsis (Nomadopsis)* collected pollen nearly exclusively from *Asteraceae* or *Rosaceae* respectively, which resulted in little overlap with other bee subgenera when considering either the identity of plants visited or the nutritional content of the pollen they collected. Less obviously, bee taxa could partition their use of taxonomically distinct host plants offering nutritionally similar pollen. For example, we frequently observed *Pyrobombus* collecting pollen from two nutritionally similar plant taxa (e.g., Onagraceae P:L 2.9; Rosaceae P:L 3.1) even though one of these (Rosaceae) was also popular with another abundant subgenera (*Nomadopsis*). Also, there was partitioning of Aster visitation where *Eumelissodes* most frequently visited *Oreostemma* and *Symphyotrichum*, while *Pyrobombus* visited *Arnica* and *Solidago* (Fig. 3, S2,3). On the other hand, although they were statistically differentiated, the two more generalized subgenera *Osmia (Melanosmia)* and *Bombus (Pryobombus)* were both more similar in terms of host plant use, with *Melanosmia* collecting pollen from a subset of *Pyrobombus*-visited plant taxa. Despite this overlap, *Pyrobombus’* frequent and somewhat unique use of higher P:L pollens resulted in a comparatively protein-enriched overall intake. It is interesting to speculate that the co-occurrence of two subgenera with relatively similar nutritional targets (*Pyrobombus* with a P:L ratio of 7.0; *Melanosmia* with a P:L ratio of 5.3) might result in a form of partitioning, where one forages on plants relatively close to its target P:L ratio (*Melanosmia*) and the other (*Pyrobombus*) achieves a similar ratio by combining visits to nutritionally imbalanced resources, often visiting both plants with the highest P:L ratios in the community (Fabaceae—*Lupinus*, Orobanchaceae—*Pedicularis*, Plantaginaceae—*Penstemon*), and those with lower P:L values (Rosaceae, Asteraceae). Whether such diversity of nutritional acquisition strategies among bees is a common or replicable phenomena remains to be seen (Behmer and Joern 2008; Machovsky-Capuska et al. 2016).

Given that we were able to detect nutritional differentiation at the subgenus level, do such patterns play out across more closely related species? Comparisons among the three most abundant *Pyrobombus* species at our site offer a more fine scale view, and more directly speak to the prospect of nutritional competition, given that we would expect more closely related taxa to have similar nutritional needs (Crisp and Cook 2012; Timberlake et al. 2024). In contrast to our other focal subgenera, *Bombus* has been the subject of several field- and laboratory-based studies exploring the nutritional ecology of pollen foraging. For example, earlier work suggested that *Bombus* generally prefers higher protein and lower lipid content in pollen (Vaudo et al. 2016a; Ruedenauer et al. 2020), and regulates its intake of these nutrients to maximize performance (Vaudo et al. 2016b). Research on *Bombus* foraging preferences among pollen host plants further shows that they routinely converge on pollen loads representing a moderate (relative to the distribution of floral pollen nutrition available), yet still high P:L niche (Vaudo et al. 2018, 2024a; Bain et al. 2025). Here, our findings are well in line with these earlier studies: we found that *Pyrobombus* occupied the highest P:L niche, and the three focal species did not differ substantially in the macronutrient content of the pollen they collected. We did resolve some subtle differences between these species that are worth considering—namely *B. flavifrons* consistently collected pollen loads with higher protein content than the other two species, which likely reflects its comparatively lower use of low P:L plants within Asteraceae or Rosaceae. This raises the question of whether *B. flavifrons* is physiologically adapted to a slightly different nutritional target, or whether it is competitively excluded from obtaining low P:L pollens that would bring its target in line with the two other species’. The similarity of collected pollen nutrition paired with taxonomic reshuffling across years would be broadly consistent with our *Bombus* species directly competing for pollen resources, but the taxonomic consistency of *B. flavifron*s’ high-protein host plant associations (*Penstemon* and *Pedicularis*) could indicate it inhabits a slightly differentiated niche (Bain et al. 2025). Manipulative experiments (e.g., such as removal of one species Brosi and Briggs 2013; Wignall et al. 2020), comparisons among sites where species do or do not co-occur, or lab-based preference performance assays (Muth et al. 2016; Vaudo et al. 2016a; Kraus et al. 2019) would be an obvious next step to differentiate between these two possibilities.

### Caveats

In this study, we delineated nutritional niches using P:L ratios. This metric is a proxy of pollen nutritional quality which has worked well to explain broad aspects of bee foraging behavior and performance in previous studies (Vaudo et al. 2016a, 2024a). We acknowledge that it is a crude description of nutritional space, as pollen is chemically complex and individual fatty acids, sterols, and specialized metabolites can also be nutritionally relevant to bees (Vanderplanck et al. 2014; Rivest and Forrest 2020; Ruedenauer et al. 2020; Arien et al. 2020). Expanding to include additional chemical traits into the nutritional niche concept is an obvious next step. It is also worth noting that we did not consider how bees are manipulating pollen via symbionts (Dharampal et al. 2019, 2022; Christensen et al. 2021) or glandular excretion (Norden et al. 1980) in ways that could change its ultimate nutritional value. Without the ability to extract pollen provisions from larval cells, our dataset can only speak to what foraging bees are collecting ahead of any further processing. Finally, among bumble bees, we only studied workers and could only make inferences based on the colony growth or reproductive rearing stage. If foundresses pursue a different nutritional target, it was not detected here as queens were rarely observed during our sample periods.

### Bee nutritional ecology beyond the models

Working within an ecological community to manipulate bee pollen resources or perform bee removal experiments is a major future challenge, which would be required to fully explore the nutritional niche concept. Yet, we should probably take on this challenge, because while lab-based manipulative experiments on model species (including our own; Vaudo et al. 2016a; Muth et al. 2018; Francis et al. 2019) are a valuable complement to field work, the external validity of this work is always an open question. In ecological communities, we expect that bee taxa differ in nutritional needs (Vaudo et al. 2024a, b), pollen resources can fluctuate (Moore and Lauenroth 2017), and competition for food is a factor (Page et al. 2024). Understanding both the nutritional needs of different bee taxa, as well as the flexibility of their realized nutritional niche in plant communities is essential for identifying what plants to conserve or restore to support wild bee populations. In this regard, our findings underline the idea that maintaining nutritional diversity could be a priority for conservation efforts: even within a relatively low diversity plant community, abundant plants at our site varied substantially in the protein and lipid content of their pollens, and we found that the pollen loads of the four most frequently observed bee subgenera occupied a wide range of P:L values. The reshuffling we observed among *Bombus* species in specific host plant use across years suggests that some of our focal taxa can pivot among alternative nutritionally similar host plants, or faced with the same host plant options, even pursue alternative strategies to achieve a similar nutritional target ratio of protein and lipids (as in *Melanosmia* and *Pyrobombus*). In addition to consideration of phenology and resource type (e.g. nectar vs. pollen), one implication of our findings is that choice of plants for conservation purposes could also be informed by consideration of pollen chemical diversity, to allow different bee taxa to occupy a variety of nutritional niches and to provide sufficient nutritional space for generalist bees’ flexibility to manifest.

## Supplementary Information

Below is the link to the electronic supplementary material.

Supplementary tables

Supplementary figures

## Data Availability

All data produced from this study are provided in the manuscript and supplementary information. All raw data are publicly available in the Forest Service Research Data Archive: Vaudo, Anthony D.; Luthy, Jillian A.; Lin, Eva; Glasser, Sonja K.; Leonard, Anne S. 2026. Wild bee host-plant pollen-visitation and bee pollen load nutritional values survey data collected from 2019–2021 in a subalpine meadow in the Sierra Nevada Mountain range. Fort Collins, CO: Forest Service Research Data Archive 10.2737/RDS-2026-0008.
