# Supplementary material for: Wild bees occupy temporally stable pollen nutritional niches
Source: Oecologia. 2026 Jul 23;208(8):98. doi: 10.1007/s00442-026-05936-z (PMC13395946; doi:10.1007/s00442-026-05936-z)
Supplement: Supplementary file 2 — Supplementary figures [file 442_2026_5936_MOESM2_ESM.pdf]

Supplementary Information:

Wild bees occupy temporally stable pollen nutritional niches

Oecologia

Anthony D. Vaudo*, Jillian A. Luthy, Eva Lin, Sonja K. Glasser, Anne S. Leonard

*Rocky Mountain Research Station, USDA Forest Service, Moscow, ID 83843
anthony.vaudo@usda.gov

Supplementary Information

**Model 1:** Bayesian model to test for differences in bee collected pollen nutrition (protein, lipid, or P:L) between bee taxa (bee subspecies or *Bombus* species).	**Model 2:** Bayesian model to test for differences in bee collected pollen nutrition (protein, lipid, or P:L) between bee taxa (bee subspecies or *Bombus* species) and years using adaptive priors between years.	**Model 3.** *Envfit* model for regressing bee pollen nutrition or bee taxa against visitation matrix ordination.	**Model 4.** Permutational multivariate analysis of variance using *adonis2* for testing differences in bee taxa or pollen nutrition against visitation distance matrices.
$nutrient \sim normal(\mu, \sigma)$<br>$\mu = \alpha_{bee\ taxa}$<br>$\alpha_{bee\ taxa} \sim normal(0, 0.5)$<br>$\sigma \sim exponential(1)$	$nutrient \sim normal(\mu, \sigma)$<br>$\mu = \alpha_{bee\ taxa} + \beta_{year}$<br>$\alpha_{bee\ taxa} \sim normal(0, 0.5)$<br>$\beta_{year} \sim normal(\bar{\beta}, \sigma_\beta)$<br>$\bar{\beta} \sim normal(0, 0.5)$<br>$\sigma_\beta \sim exponential(1)$<br>$\sigma \sim exponential(1)$	a. envfit(<br>rda(<br>decostand(<br>visitation_matrix,<br>method =<br>“normalize”))<br>~ bee_taxa,<br>data = environment,<br>display = "sites")<br>b. envfit(<br>rda(<br>decostand(<br>visitation_matrix,<br>method =<br>“normalize”))<br>~ protein + lipid + P:L,<br>data = environment,<br>display = "sites")	a. adonis2(<br>formula =<br>decostand(<br>visitation_matrix,<br>method =<br>“normalize”))<br>~ bee_taxa,<br>data = environment,<br>permutations = 1000,<br>by = "terms",<br>strata = year)<br>b. adonis2(<br>formula =<br>decostand(<br>visitation_matrix,<br>method =<br>“normalize”))<br>~ protein + lipid + P:L,<br>data = environment,<br>permutations = 1000,<br>by = "terms",<br>strata = year)

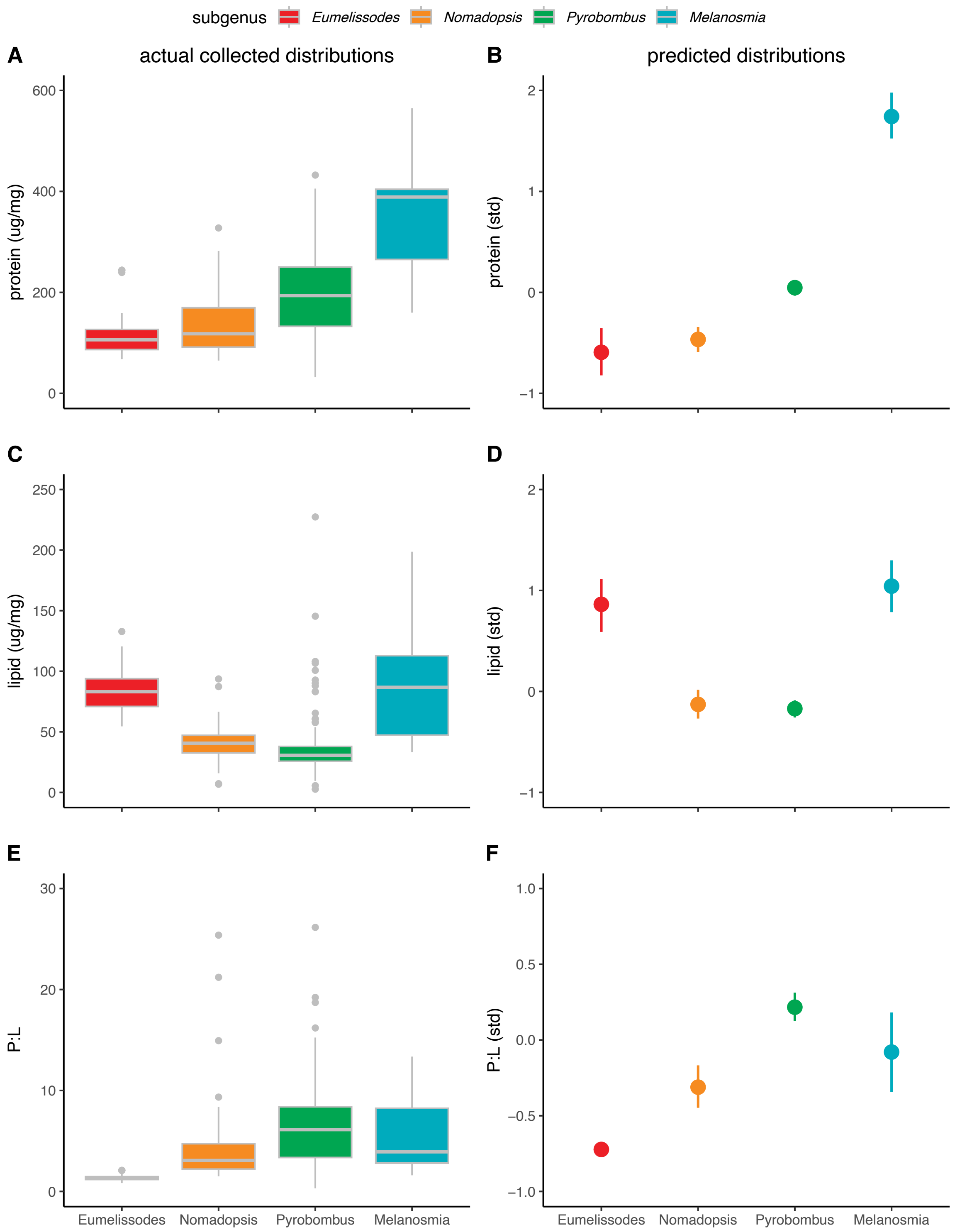

Figure S1. Bee subgenus pollen nutrition distributions across all years. Boxplots (A,C, and E) are actual distributions with median, 1st and 3rd quartiles and whiskers 1.5*IQR. Panels B,D, and F are Bayesian posterior predicted mean and 90% credible intervals, indicating predicted values of nutritional composition of each bee taxa.

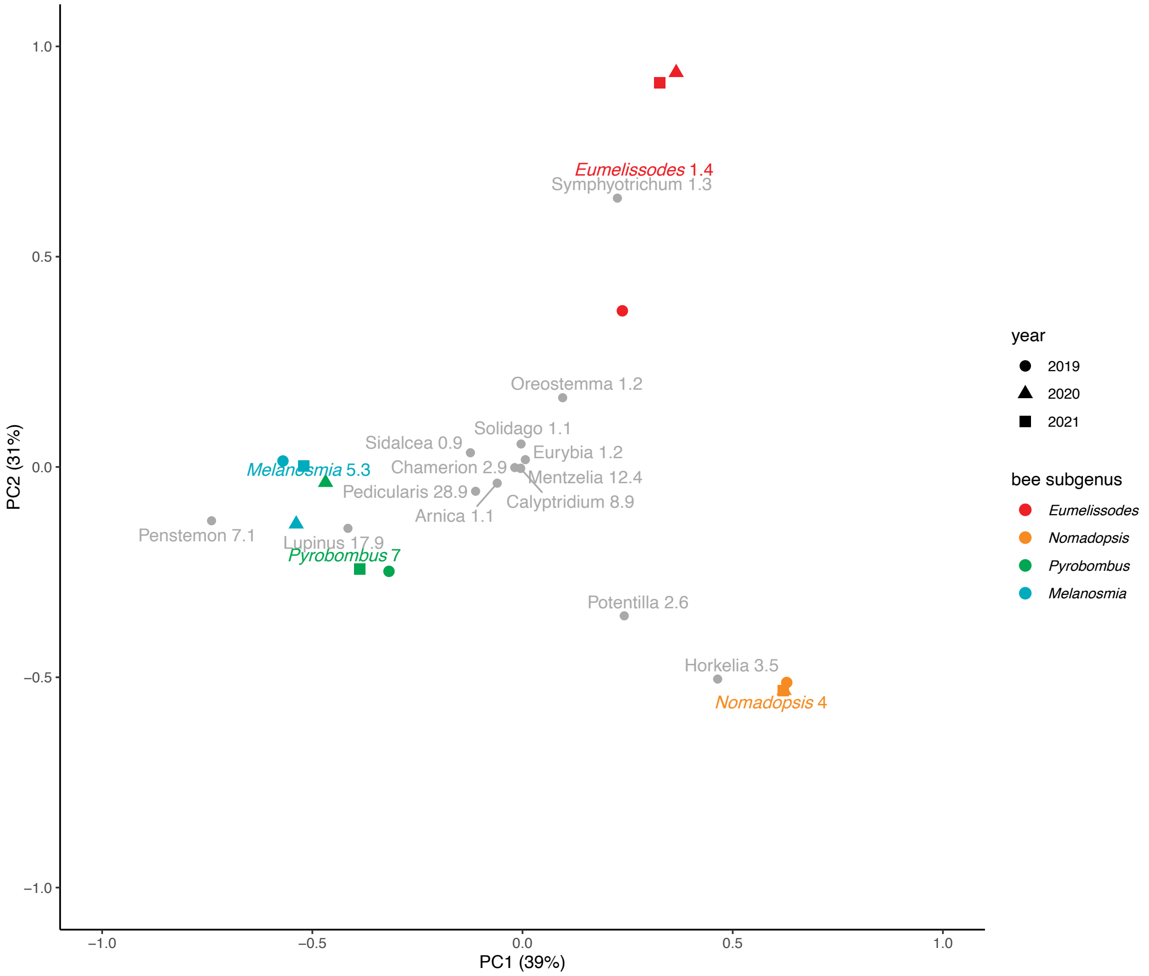

Figure S2. Principal components (PCA) plots of bee subgenus visitation to plant genera (color = bee subgenus; shape = year). Labels indicate average P:L values of pollen loads measured in this study, or floral pollen of plant taxa present at site (from Vaudo et al. 2024a).

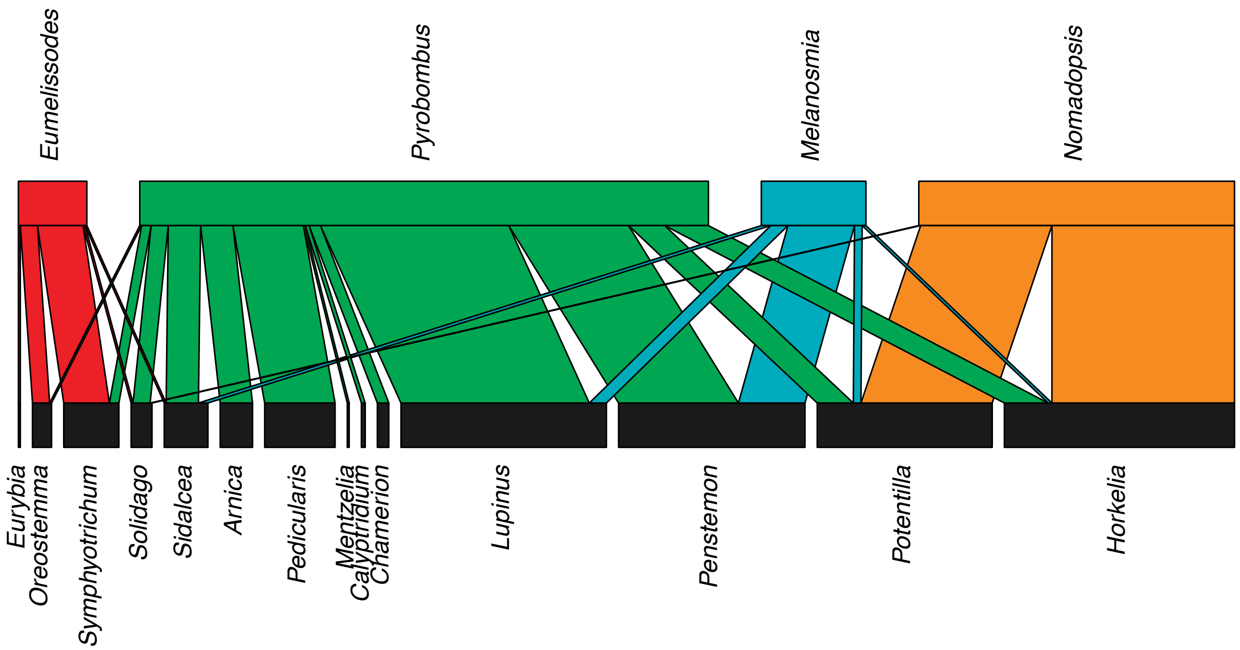

Figure S3. Interaction metanetwork of bee subgenera and plant genera across all three years. Width of bee taxa = relative numbers observed; width of plant taxa = relative numbers of visits observed; width of connectors = relative visitation frequency.

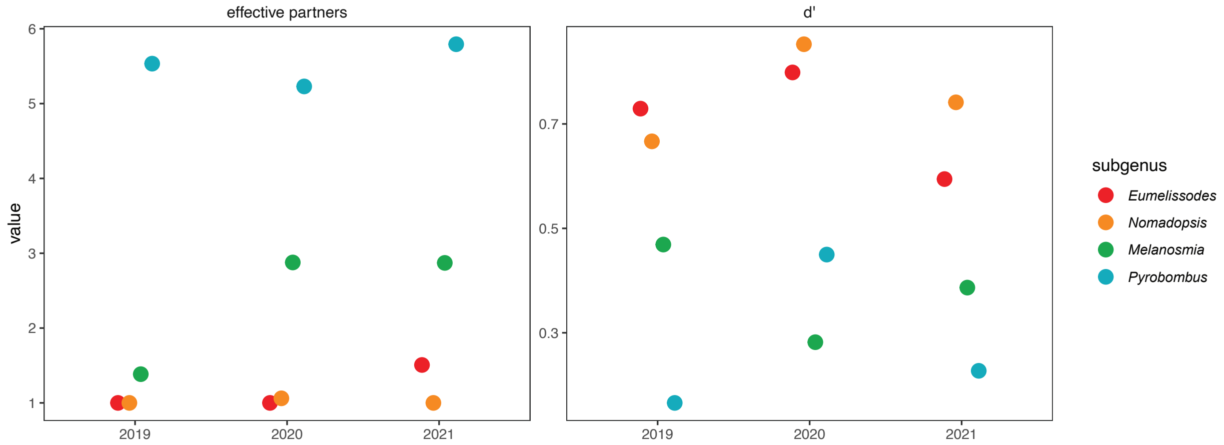

Figure S4. Indices of foraging specialization of bee subgenera to plant genera across years. Lower *effective partners* and higher *d'* values indicate higher levels of specialization.

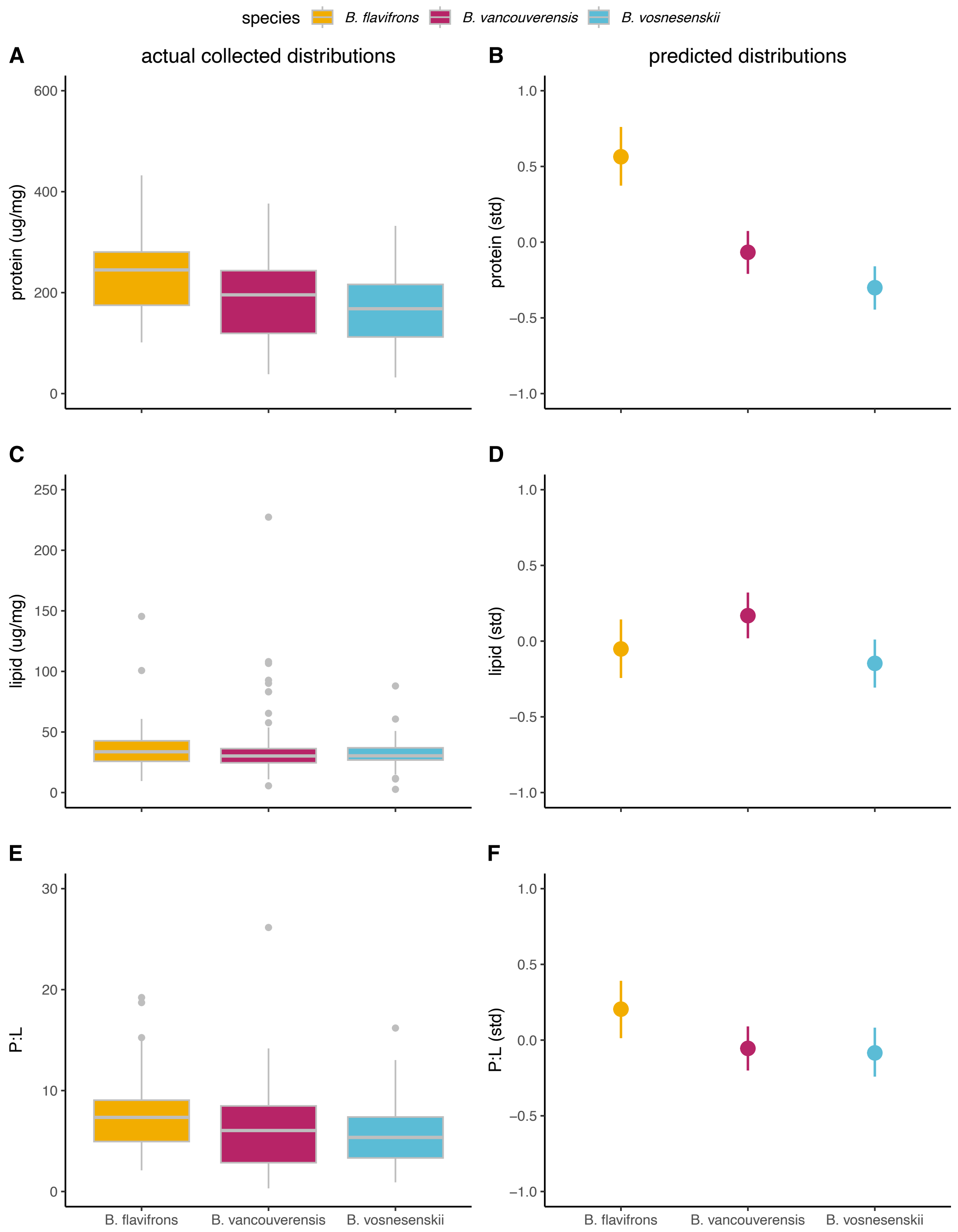

Figure S5. *Pyrobombus* species pollen nutrition distributions across all years. Boxplots (A,C, and E) are actual distributions with median, 1st and 3rd quartiles and whiskers 1.5*IQR. Panels B,D, and F are Bayesian posterior predicted mean and 90% credible intervals, indicating predicted values of nutritional composition of each species in each year.

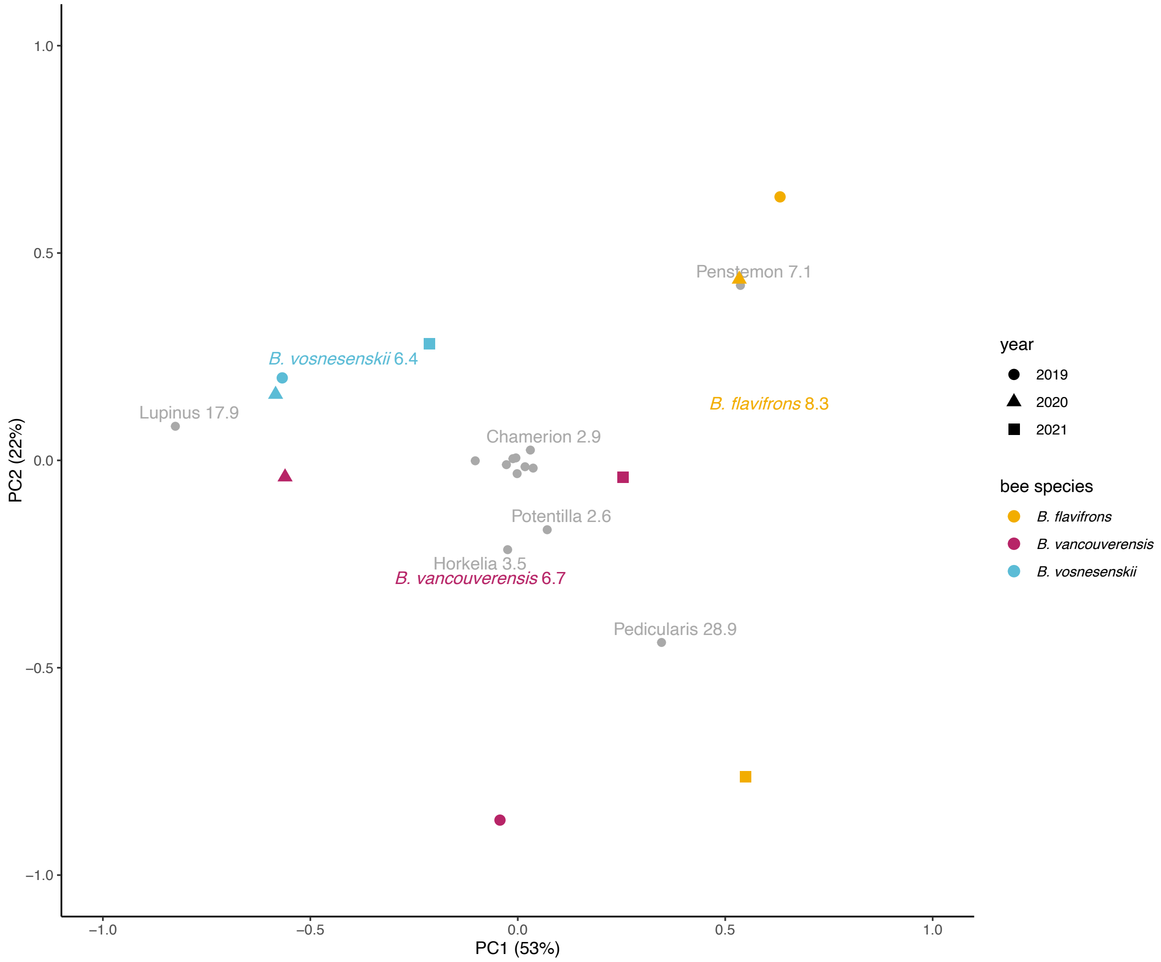

Figure S6. Principal components (PCA) plots of *Pyrobombus* species visitation to plant genera (color = species; shape = year). Labels indicate average P:L values of pollen loads measured in this study, or floral pollen of plant taxa present at site (from Vaudo et al. 2024a).

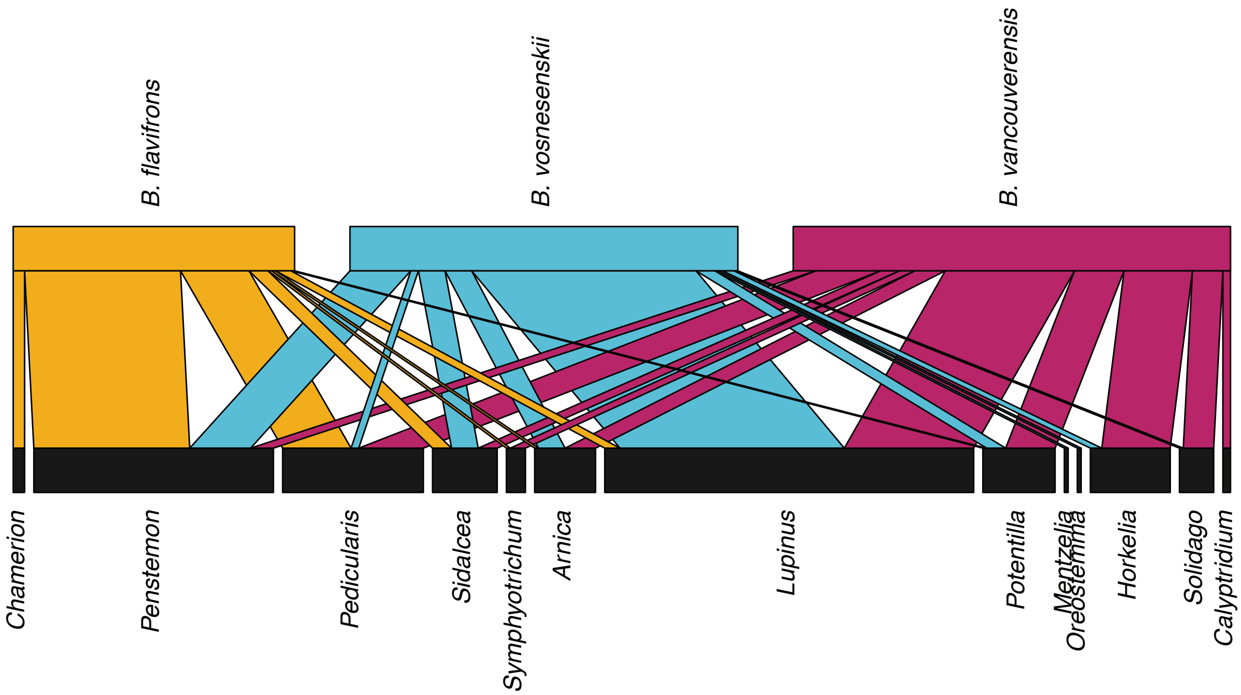

Figure S7. Interaction metanetwork of *Pyrobombus* species and plant genera across all three years. Width of bee taxa = relative numbers observed; width of plant taxa = relative numbers of visits observed; width of connectors = relative visitation frequency.

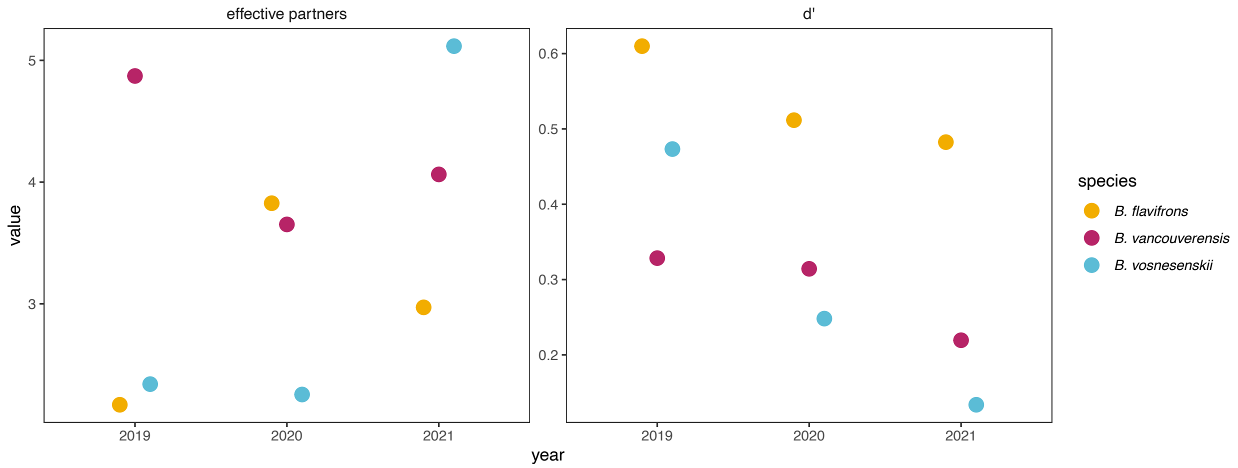

Figure S8. Indices of foraging specialization of *Pyrobombus* species to plant genera across years. Lower *effective partners* and higher *d'* values indicate higher levels of specialization.
